# Activity of hypertonic solution with Silver and Potassium Sucrose Octasulfate on nasal symptoms in obstructive rhinopathy with and without rhinosinusitis

**DOI:** 10.1186/2193-1801-2-668

**Published:** 2013-12-13

**Authors:** Desiderio Passali, Jacopo Cambi, Francesco Maria Passali, Luisa Bellussi

**Affiliations:** Dipartimento di Scienze neurologiche e sensoriali, Università di Siena, Viale Bracci, Siena, 11 53100 Italy; Dipartimento di Chirurgia, Università di Roma Tor Vergata, Viale Oxford 81, Roma, 00133 Italy

**Keywords:** Nasal obstruction, VAS, SNOT-22, Rhinopathy, Silver sucrose octasulfate, Potassium sucrose octasulfate

## Abstract

**Background:**

Nasal obstruction is a primary symptom of common upper respiratory tract disorders. In clinical practice nasal saline solutions are recommended for the cleansing of nasal cavities and relieving nasal symptoms.

**Methods:**

55 patients (aged 25–70 years) suffering from obstructive rhinopathy, with nasal obstruction/congestion of moderate severity persistent since at least 10 days in advance of recruitment with/without rhinosinusitis was randomly treated with an hypertonic solution composed by Silver Sucrose Octasulfate and Potassium Sucrose Octasulfate (SILSOS) or isotonic solution for 20 days.

At baseline (T0), ten days (T10) and twenty days (T20) after SILSOS treatment, study participants were evaluated subjectively with VAS and SNOT-22, objectively by Active Anterior Rhinomanometry (AAR) and MCC/MCTt determination. Forty-four patients were followed-up 30 days after the end of treatment by a phone interview.

**Results:**

The AAR analysis showed in SILSOS group a significantly (p < 0.05) ameliorated in expiratory flow, at T0-T10 and T0-T20. No improvement in MCTt was observed over the 20 days study period. The mean values MCC of significantly improved at T20 (p < 0.05). VAS total score showed improvement along all time-intervals. Nasal obstruction was back 30 days after the end of treatment with SILSOS in only 3 patients and reported to be in a mild form.

**Conclusions:**

The obtained results show that SILSOS hyper has added to the mechanical action of removal of secretions a specific decongestant and antiseptic effect lasting longer after the end of treatment. Could help to fluidize thick mucus, improve respiration and promote resolution of symptoms, preventing pathogens adhesion to nasal mucosa.

## Background

The complaint of blockage, fullness, or restricted airflow are frequent in patients with nasal obstruction. The mucosal inflammation and decreased nasal patency, are common condition in cold and acute or chronic rhinosinusitis with/without nasal polyposis.

Rhinosinusitis are defined as inflammation of the nose and the paranasal sinuses characterized by two or more symptoms, one of which should be either nasal blockage obstruction congestion or nasal discharge (anterior/posterior nasal drip) ± facial pain/pressure or ± reduction or loss of smell (Fokkens et al. 2007; Rosenfeld et al. 2007; Scadding et al. 2008). Anterior Active Rhinomanometric (AAR) measurement of nasal resistance and nasal peak flow correlate well with subjective sensation of nasal obstruction (Fokkens et al. 2012; Passali et al. 2000), although this correlation remain uncertain (Andre et al. 2009). The AAR provides an objectively value of left, right, and total Nasal Airflow Resistance (NAR, NAR_total_ = NAR_left_ × NAR_right_/NAR_left_ + NAR_right_ (Clement 1991))_._

A 10-cm Visual Analogic Scale (VAS) was validated for patients with rhinosinusitis and divided the severity of disease into mild (VAS 0–3), moderate (VAS >3-7) and severe (VAS >7-10) (Fokkens et al. 2007; Scadding et al. 2008; Fokkens et al. 2012). A VAS > 5 affects patients Quality of Life (QOL) (Lim et al. 2007).

Sino-Nasal Outcome Tests (SNOT-22) (Hopkins et al. 2009) was an health related quality of life instrument for chronic rhinosinusitis and range from 0 (absence of symptoms) to 5 (the highest severity degree).

Mucociliary clearance (MCC) and/or the mucociliary transport time (MCTt) are involved in the defensive mechanisms against paranasal sinuses infection, in patients with rhinosinusitis assume pathological values due transudation that thickening the periciliary layer (Antunes et al. 2009; Cohen 2006; Passali 2003; Jones 2001).

MCC and MCTt were determined by means of the charcoal + 3% saccharine test. The insoluble charcoal powder determinate the transport of foreign bodies like bacteria or dust particles entrapped into the outer mucus layer, the soluble saccharine measure the clearance (i.e. the dilution and drainage) of solutes into the inner mucus layer. The patient’s perception of the sweet saccharine taste and the black color of charcoal in pharynx are easily detectable. MCC/MCTt is reported to take more than 30 minutes in pathological conditions; MCTt normal values are 13 ± 2 minutes in adults; MCC normal values are 17 ± 5 minutes in adults (Passali et al. 1984).

Endoscopic signs of rhinosinusitis are polyps and/or mucopurulent discharge primarily from middle meatus and/or oedema/mucosal obstruction primarily in middle meatus, that promote stasis of secretions and proliferation of bacteria to on sinonasal mucosa, bringing to an inflammatory mucosal response, worsening sinonasal symptoms and facilitating onset and/or recurrence of infections. Consequently, the solubilisation and release of secretions represent a basis of symptom resolution and functional improvement. Isotonic and hypertonic saline nasal wash/irrigation are an effective intervention, adjunct or not to medical therapy, to determinate MCC/MCTt recovery, reduce nasal mucosal edema, and make easy the elimination of microorganisms and secretions (Harvey et al. 2007; Thomas et al. 2008; Süslü et al. 2009; Talbot et al. 1997).

In patients with acute upper respiratory tract infections saline nasal irrigation is associated with less time off work and with a tendency towards less antibiotic usage (Kassel et al. 2010).

SILSOS hyper, CM&D Pharma Limited is a Medical Device (MD) composed by the association of Silver Sucrose Octasulfate (IASOS; US7183315, EP1458733) and Potassium Sucrose Octasulfate (KSOS). Combining the antimicrobial property of IASOS and the carbohydrate-based microbial antiadhesion of KSOS, SILSOS hyper restore nasal mucosae, reinforce the tropism through Fibroblast Growth Factor pathway activation and facilitate mucosal decongestion and hydration (Rashid et al. 1999; Yeh et al. 2002).

The main aim of this study was to valuated the efficacy of a 20 days period of treatment with MD on MCC and MCTt values compared to the simple treatment with isotonic solution. Valuate usefulness on primary and secondary symptoms of rhinosinusitis using visual analog scale (VAS) and Sino-Nasal Outcome Tests (SNOT-22) in patients with nasal respiratory obstruction.

## Methods

Between February 2012 and February 2013, 55 consecutive patients (aged 25–70, 20 male) with obstructive rhinopathy of various aetiology were seen in our Department. Inclusion criteria was have persistent symptoms since at least 10 days in advance of recruitment due to inferior turbinate hypertrophy or congestion associated at a pre-existing deviated nasal septum, VAS > 5 for two of the primary symptoms: nasal congestion, nasal obstruction and Rhinorrea or VAS > 5 for one of the primary symptoms above and VAS >3 for at least one of the secondary symptoms: facial pain/pressure, reduction or loss of smell. Exclusion criteria were pregnancy, persistent/intermittent allergic rhinitis, cystic fibrosis, gross immunodeficiency (congenital or acquired), congenital mucociliary problems, fungal disease, systemic vasculitis and granulomatous diseases, cocaine abuse, diagnosis of nasal polyps (Lund/McKay II-III degree), nasal neoplasia; participation in other clinical trials within 3 months from enrolment; treatment with local and/or systemic corticosteroid, antibiotic, decongestants and nasal saline washes within one week from enrolment.

The patients were randomly assigned by a computer-generated eight blocks code to treatment by isotinic solution (n = 30) or MD treatment (n = 25).

Participants were evaluated subjectively with VAS and SNOT-22, and objectively with AAR, MCC and MCTt at baseline (T0), ten days (T10) and twenty days (T20) after MD treatment (2 sprays/nostril, two times a day for 20 days).

To assess compliance, the dispenser weight at T0 and at T20 were recorded.

All the patients were followed-up after 30 days the end of the treatment with a phone interview aimed to evaluate the long term effectiveness of the treatment.

After receiving detailed information about study aim, all participants signed their Informed Consent in compliance with the Declaration of Helsinki and current Good Clinical Practice. The study protocol was approved by the local Ethics Committee of the University Hospital “Le Scotte”, Siena (Nr 93/2011, November 22, 2011).

ANOVA was performed on continuous variables and categorical variables. Kruskal-Wallis were applied as appropriate for significance between treatment groups (Saline vs MD). Comparisons between groups were assessed by the Wilcoxon Signed Rank Test (significance of changes in secondary endpoints at different time intervals) or Friedman Test or *t*-test, as appropriate, at a significance level of p < 0.05. Statistical analysis was performed with SPSS software (SPSS, Inc., Chicago, IL, USA).

## Results

Twenty patients had acute rhinosinusitis at the beginning of the therapy with MD, these patients have associated at the nasal irrigation an antibiotic therapy (500 mg/die of fluoroquinolone drug class) for 14 days.

Controls of dispenser’s weight does not have shown a lack of compliance by patients who have received treatment with MD while in 5 patients in the saline solution group there was a remaining weight more than 25% of the total at T1examination and therefore these patients were excluded from the study and from results.

AAR analysis were reported in Table 1, at baseline total NAR were more than doubled the healthy 0,25 Pa/ml/s reported value (Clement 1991). MD progressively and significantly (p < 0.05, Wilcoxon signed rank test) ameliorated in expiratory flow, both at T0-T10 and T0-T20. No significantly variation was observed in control group.

**Table 1:** **AAR. Total nasal resistance at different timepoints**

	MD - Total resistance (Pa/ml/s)	
Time	Inspiratory flow	Expiratory flow
**T0**	0.660 ± 0.601	0.662 ± 0.579
**T10**	0.510 ± 0.427	0.470 ± 0.327*
**T20**	0.420 ± 0.218	0.382 ± 0.205*

*p < 0.05 versus T0, Wilcoxon signed rank test.

Table 2 report MCTt and MCC values at baseline, and after ten (T10) and twenty (T20) days of treatment. The mean value of MCTt at T0 was normal while MCC times were at the upper limits of the normal range. No significant improvement in MCTt was observed over the 20 day study period, or at T0-T10 and T10-T20 timepoints. The mean MCC values significantly improved at T20 (p = 0.0003, Friedman test), the ΔT0-T20 was 4.12 minutes. These differences have to be considered clinically significant. No significantly variation was observed in control group.

**Table 2:** **MCTt and MCC at T0, T10 and T20 days after treatment. Data express the mean ± SD**

	MCTt (minutes)	P (Friedman test)	MCC (minutes)	P (Friedman test)
**T0**	13.0 ± 1.10	Vs T10 > 0.05	20.20 ± 4.38	Vs T10 > 0.05
		Vs T20 > 0.05		Vs T20 < 0.05*
**T10**	12.64 ± 0.81	Vs T20 > 0.05	17.96 ± 4.23	Vs T20 > 0.05
**T20**	12.72 ± 0.84	/	16.08 ± 4.24	/

*p < 0.05 T0 versus T20, Friedman test.

Table 3 reports VAS total score (mean ± SD) for primary, primary plus secondary symptoms, and the VAS Mean Score (mean ± SD), defined as: total symptoms score/n. symptoms, and expressing the severity of the reported symptoms. On the overall treatment period (T0-T20), MD improved VAS total score for primary, primary and secondary symptoms, and VAS mean score (p < 0.05 vs T0). For primary and secondary symptoms MD showed a VAS total improvement along all time-intervals (p < 0.05). In general, the MD demonstrated to be as effective on MCC times and better scores in primary, primary and secondary symptoms, and their severity degree at T0-T20. Similar variation in VAS total score (mean ± SD) for primary, primary plus secondary symptoms, and the VAS Mean Score was reported at T20 in isotonic control group.

**Table 3:** **VAS Total and Mean Score (mean ± SD) at different timepoints in the two study groups**

	Primary symptoms		Primary + secondary symptoms		Total score/n symptoms	
	VAS total score		VAS total score		VAS mean score	
		P (Friedman test)		P (Friedman test)		P (RM ANOVA)
**T0**	11,5 ± 3,4	Vs T10 < 0.05*	15.6 ± 3.7	Vs T10 < 0.05*	5,7 ± 1,5	Vs T10 > 0.05
		Vs T20 < 0.001**		Vs T20 < 0.001**		Vs T20 < 0.05°
**T10**	9,6 ± 3,1	Vs T20 > 0.05	12.8 ± 3.5	Vs T20 < 0.05°°	4,9 ± 1,3	Vs T20 > 0.05
**T20**	7,6 ± 2,8		10.5 ± 4.0		4,0 ± 1,3	

*p < 0.05 T0 versus T10, Friedman test; **p < 0.001 T0 versus T20, Friedman test; °p < 0.05 T0 versus T20, Repeated Measures ANOVA; °°p < 0.05 T10 versus T20, Friedman test.

Table 4 reported the effects of MD on the SNOT-22 scores, after twenty days of use an improvement in nasal obstruction/congestion, posterior nasal discharge and thick nasal discharge items was observed with an improvement in productivity and concentration items. In control group was detected an improvement in total SNOT-22 scores, in nasal obstruction/congestion item and in posterior nasal discharge.

**Table 4:** **Effect of the MD on the SNOT-22 Scores (Median values)**

ITEMS	MD		
	T0	T10	T20
Need to blow the nose	2,12 ± 1,0 (2)	2,28 ± 1,0 (2)	2,04 ± 1,0 (2)
Sneezing	1,40 ± 1,2 (1)	1,08 ± 0,9 (1)	0,88 ± 0,9 (1)
Runny nose	2,00 ± 1,6 (2)	2,00 ± 1,5 (2)	2,00 ± 1,3 (2)
Cough	0,76 ± 1,0 (0)	0,52 ± 0,8 (0)	0,44 ± 0,9 (0)
Posterior nasal discharge	1,68 ± 1,5 (1)	1,16 ± 1,1 (1)	0,60 ± 0,8* (1)
Thick nasal discharge	1,28 ± 1,1 (1)	0,72 ± 0,7 (1)	0,56 ± 0,8* (1)
Ear fullness	1,40 ± 1,0 (1)	1,20 ± 0,7 (1)	0,92 ± 0,5* (1)
Dizziness	------------	------------	------------
Ear pain	0,12 ± 0,4 (0)	0,08 ± 0,3 (0)	0,00 ± 0,0 (0)
Facial pain/pressure	1,12 ± 1,5 (1)	0,84 ± 1,3 (1)	0,76 ± 1,2 (1)
Difficulty falling asleep	1,80 ± 1,5 (2)	1,60 ± 1,3 (2)	1,60 ± 1,3 (2)
Waking up at night	1,90 ± 1,3 (2)	1,40 ± 1,2 (1)	1,40 ± 1,2 (1)
Lack of a good night sleep	2,10 ± 1,4 (2)	1,60 ± 1,3 (2)	1,60 ± 1,2 (2)
Waking up tired	2,00 ± 1,4 (2)	1,80 ± 1,1 (2)	1,80 ± 1,2 (2)
Fatigue	1,90 ± 1,4 (2)	1,70 ± 1,2 (2)	1,40 ± 1,3 (2)
Reduced productivity	0,80 ± 1,1 (1)	0,64 ± 1,1 (0)	0,16 ± 0,5**° (0)
Reduced concentration	1,20 ± 1,0 (1)	0,64 ± 1,0* (1)	0,40 ± 0,9** (0)
Frustrated/restless/irritable	0,60 ± 1,0 (0)	0,36 ± 0,9 (0)	0,40 ± 1,1 (0)
Sad	0,04 ± 0,2 (0)	0,04 ± 0,2 (0)	0,12 ± 0,6 (0)
Embarrassed	0,04 ± 0,2 (0)	0,08 ± 0,3 (0)	0,12 ± 0,6 (0)
Sense of smell/taste	1,48 ±1,6 (1)	0,88 ± 1,4 (1)	0,68 ± 1,2 (0)
Nasal obstruction/congestion	4,64 ± 0,5 (4)	3,72 ± 0,8** (3)	2,96 ± 0,9**^Δ^ (3)
TOTAL SCORE	30,0 ± 9,7 (28)	24,2 ± 8,4* (24)	20,8 ± 8,7** (21)

T20 Vs T0 : *p < 0,05 **p < 0.001; T20 Vs T10 : °p < 0,05, ^Δ^p < 0. 005 (Wilcoxon Test).

**Figure 1:**
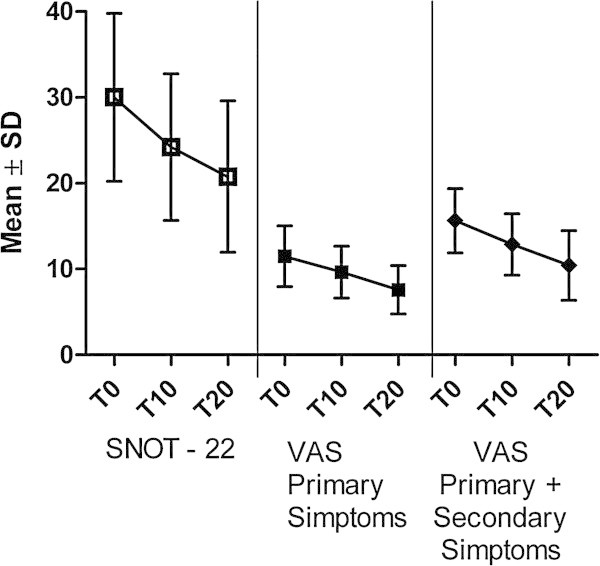
**SNOT-22, VAS Primary Simptoms and VAS Primary + Secondary Simptoms variation at T10 and T20.** On the overall treatment period (T0-T20), MD improved VAS total score for primary, primary + secondary symptoms, and SNOT-22 mean score (p < 0.05 vs T0).

MD effect on nasal obstruction/congestion and nasal discharge, being these symptoms of relevance in the clinical diagnosis of rhinosinusitis, were also considered in the small, good balanced subgroup of rhinosinusitis patients. Results are summarized in Figure 1.

All data about the control group was reported in Table 5.

**Table 5:** **Control group data**

Isotonic solution	T0	T10	T20
Inspiratory flow (Pa/ml/s)	0.533 ± 0.439	0.507 ± 0.336	0.486 ± 0.207
Expiratory flow (Pa/ml/s)	0.542 ± 0.435	0.502 ± 0.285	0.475 ± 0.212
MCTt (minutes)	13.0 ± 1.3	12.88 ± 0.78	12.84 ± 0.86
MCC (minutes)	21.52 ± 4.593	19.68 ± 4.498	19.60 ± 5.480
Primary symptoms	11,2 ± 3,7	8,6 ± 2,7*	7,1 ± 3,2**
Primary + secondary symptoms	15.5 ± 4.5	12.4 ± 4.0*	10.5 ± 4.1**
VAS Mean score	5,6 ± 1,7	4,7 ± 1,3	4,0 ± 1,5**
SNOT-22 Total score	33,0 ± 12,0	26,0 ± 10,0*	22,0 ± 11,0**

*T0 Vs T10 < 0.05 ; **T0 Vs T20 < 0.05.

44/50 patients carried the follow up-interview, 30 days after treatment, only 3/22 patients of MD group reported the presence of subjective nasal obstruction, which was graded as a mild form. In control group 10/22 patients after 30 days reported the presence of subjective nasal obstruction.

## Discussion

In our study population, nasal obstruction was the most relevant patients’ disorder as well as a major inclusion criteria to enrollment. After treatment, the more salient effect observed was the significantly decreased scoring for nasal obstruction/congestion on the overall treatment period. The symptom relief resulted to depend upon amelioration of posterior nasal discharge, thick discharge and ear fullness. These parameters suggest a success in fluidification of sinonasal secretions, supporting the recovery on health-related quality of life.

Although subjective assessment of nasal obstruction by patient-reported outcome measurements (PROMs) is a well-validated criterion, if little correlation were found between a patient-based symptom severity-scoring systems and an objective respiratory parameter, the impact of symptom amelioration could be overestimated. In our patients, a good matching of ameliorated PROMs and total nasal resistance was observed at T20, indicating an improved respiration.

Subjective improvement that was observed in the VAS scores and in SNOT 22 scores both in subjects treated with MD than in control group was not confirmed by the rhinometry data that showed an objective statistically significant improvement only in the MD group.

Rhinomanometry has been reported to correlate with subjective symptom scoring with and without decongestion (Eccles et al. 2005). There is an excellent correlation, considering studies with normal controls, patients with structural abnormalities, hyper-reactivity or infective rhinitis, between the subjective sensation of nasal obstruction and AAR values (Fairley et al. 1993; Sipilä et al. 1994; Simola and Malmberg 1997; Hirschberg and Rezek 1998; Numminen et al. 2003; Nathan et al. 2005). Even if some reports did not validate these data (Jones et al. 1989) or showed weak associations between PROMs and rhinomanometry (Eccles and Jones 1983; Roithmann et al. 1994; Panagou et al. 1998), does not necessarily imply that either subjective or objective scores are invalid, because these two approaches measure different aspects of the disease process. Subjective nasal obstruction correlates better with objective functional measurements of nasal airflow resistance (rhinomanometry, peak flow) than with measurements of nasal cavity width, such as acoustic rhinometry (Numminen et al. 2003; Szücs and Clement 1998). The measurement of nasal airway resistance by assessing nasal flow at a constant pressure can be useful in confirming that improvement in nasal congestion is the result of reduction in inflammation in the middle meatus rather than mechanical obstruction.

There is a limitation in main aim of the study because upon inclusion, patients had normal MCTt values and MCC times were at the upper limits of the normal range. Hence, no large improvements could be expected from a one to two week course of treatment. Considering these baseline values, the observed improvement in MCC can indeed be interpreted as clinically convincing. The greater success of the MD on the MCC times could depend on its hyper osmolarity. Hypertonic solutions are more helpful than isotonic solution since the drainage of the solutes into the inner “sol” layer can benefit of the dilution induced by the osmotic effect (Rashid et al. 1999). MCTt is expression of the equilibrium between both the inner “sol” layer and the outer “gel” layer and therefore, it needs prolonged or repeated treatments before a change could be appreciated.

An interesting findings comes from self-reported recurrences at follow-up. Nasal obstruction was back 30 days after the end of treatment in only 3 patients, and reported to be in a mild form. MD has the mechanical action of removal secretions, a specific decongestant and antiseptic effect lasting longer after the end of treatment.

MD could help to fluidize thick mucus, improve respiration and promote resolution of symptoms, in view of its natural decongestant activity and of its hydrating effects. MD were very well tolerated by patients since no adverse effect or complaints was recorded during the study, compliance was 78%.

The absence of Sodium Chloride in MD exclude the burning and bleeding events, sometime referred for nasal physiological solutions. The new MD seems to represent a secure alternative to present nasal salty preparations, alone or in adjunct to the medical therapy, with the advantage of a superior symptom relief.

## Conclusions

This paper describes the results obtained by patients with acute upper respiratory tract infections or obstructive rhinopathy from nasal irrigation with SILSOS hyper. At the mechanical action of removal secretions adds a specific decongestant and antiseptic effect lasting longer after the end of treatment which result in an improvement in PROMs like VAS and SNOT-22. Could help to fluidize thick mucus, improve respiration and promote resolution of symptoms, preventing pathogens adhesion to nasal mucosa.

## Abbreviations

(AAR): Anterior Active Rhinomanometric
(NAR): Nasal Airflow Resistance
(VAS): Visual Analogic Scale
(QOL): Quality of Life
(SNOT-22): Sino-Nasal Outcome Tests
(MCC): Mucociliary Clearance
(MCTt): Mucociliary Transport time
(MD): Medical Device
(IASOS): Silver Sucrose Octasulfate
(KSOS): Potassium Sucrose Octasulfate
(PROMs): Patient-Reported Outcome Measurements.
